# Genetic Characterization of a Natural Reassortant H3N8 Avian Influenza Virus Isolated from Domestic Geese in Guangxi, Southern China

**DOI:** 10.1128/genomeA.00747-14

**Published:** 2014-07-31

**Authors:** Tingting Liu, Zhixun Xie, Degui Song, Sisi Luo, Liji Xie, Meng Li, Zhiqing Xie, Xianweng Deng

**Affiliations:** a College of Life Science, Guangxi Normal University, Guilin, Guangxi Province, China; b Guangxi Key Laboratory of Animal Vaccines and Diagnostics, Guangxi Veterinary Research Institute, Nanning, Guangxi Province, China

## Abstract

A H3N8 subtype of avian influenza virus, A/goose/Guangxi/020G/2009(H3N8) (GX020G), was isolated from the Guangxi Province of China in 2009. All eight gene segments of the GX020G strain were sequenced. Sequence analysis indicated that this H3N8 virus is a novel reassortant strain. The genome sequences provide useful information for understanding the epidemiology and molecular characteristics of the H3N8 subtype of influenza virus in southern China.

## GENOME ANNOUNCEMENT

The avian influenza virus (AIV) is a negative-sense, segmented RNA virus that belongs to the genus Influenza type A virus of the family *Orthomyxoviridae* (1, 2). There are 17 hemagglutinin (HA) and 10 neuraminidase (NA) subtypes of AIV based on the antigenic differences of the HA and NA proteins, which are surface glycoproteins on the viral envelop (3–5). This H3 subtype of AIV belongs to low pathogenic AIV (LPAIV), which is one of the predominant subtypes among LPAIVs. Some studies indicated that the H3 subtype has a high separation rate and the seasonal variations in the isolation of the H3 subtype of AIV are consistent with those of human H3 subtype influenza viruses (6, 7). Research studies also predicts that the H3 subtype of AIV may have the ability to cross the species barrier to infect humans through gene reassortment (8, 9). Moreover, the Hong Kong influenza virus (H3N2) in 1968 was a reassortant with avian (H3) PB1 and HA genes and six other genes from the human (H2N2) virus (10). Therefore, it is necessary to enhance the surveillance of the H3 subtype of AIV.

In this study, A/goose/Guangxi/020G/2009 (H3N8) was isolated from a goose in a live poultry market in Guangxi, China, in 2009. The eight genes were amplified by real-time PCR using AIV universal primers (11–13). The amplified products were purified and cloned into the pMD-18T vector (TaKaRa) and sequenced (TaKaRa, Dalian, China). Sequences were assembled and manually edited to generate the final full-length genome sequence.

The complete genome of the GX020G strain consists of eight gene segments of PB2, PB1, PA, HA, NP, NA, M, and NS genes. The full lengths of these segments are 2,341, 2,341, 2,233, 1,765, 1,565, 1,460, 1,027, and 890 nucleotides, respectively. The amino acid residues at the cleavage site (340–348) of the HA molecule are PEKQTR↓GLF with one basic amino acid, which is characteristic of low pathogenic AIV. The PB2 protein possesses E627 and D701, which is characteristic of AIV. The M2 protein possesses S31, which is not amantadine resistant. The amino acid residues at the receptor binding site in the HA protein are Q226 and G228, different than L226 and S228 in the H3 subtype of human influenza viruses, which preferentially bind to an avian-origin receptor.

Sequence analysis indicates that the nucleotide sequences of both HA and NA genes of the GX020G strain both belong to the Eurasian lineage. The HA gene shows the highest homology (95%) to that from A/duck/Beijing/40/2004 (H3N8). The NA gene shows the highest homology (98.1%) to that from A/swine/Guangdong/K4/2011 (H4N8), which suggests these strains may share similar original ancestors. The other genes show the highest homology (≥97%) to some Eurasia subtypes.

These data indicates that the GX020G is a novel reassortant virus whose genes derived from multiple AIV strains, and the genome information of GX020G is helpful in conducting epidemiology investigation on the H3N8 subtype of AIV in China.

### Nucleotide sequence accession numbers.

The genome sequence of A/goose/Guangxi/020G/2009 (H3N8) has been deposited at GenBank under the accession no. KJ764713 to KJ764720.
